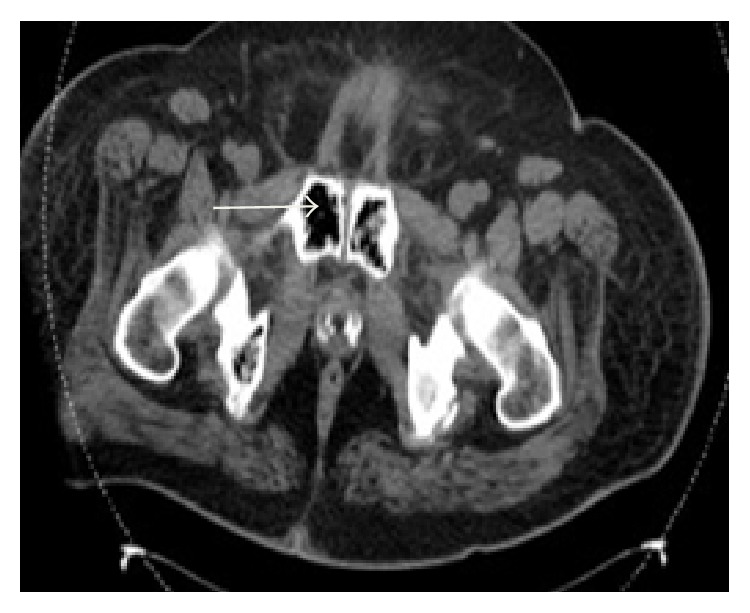# *Clostridium septicum* Gas Gangrene in Colon Cancer: Importance of Early Diagnosis

**DOI:** 10.1155/2015/694247

**Published:** 2015-12-17

**Authors:** Sowmya Nanjappa, Sweta Shah, Smitha Pabbathi

**Affiliations:** ^1^Department of Internal Hospital Medicine, H. Lee Moffitt Cancer Center, Tampa, FL 33612-9416, USA; ^2^USF College of Medicine, 12902 Magnolia Drive, Tampa, FL 33612-9416, USA; ^3^Internal Medicine Residency Program, University of South Florida College of Medicine, 12902 Magnolia Drive, Tampa, FL 33612-9416, USA

## Abstract

The Clostridia species are responsible for some of the deadliest diseases including gas gangrene, tetanus, and botulism.* Clostridium septicum* is a rare subgroup known to cause atraumatic myonecrosis and is associated with colonic malignancy or immunosuppression. It is a Gram-positive, anaerobic, spore-forming bacillus found in the gastrointestinal tract and can lead to direct, spontaneous infections of the bowel and peritoneal cavity. The anaerobic glycolysis of the tumor produces an acidic, hypoxic environment favoring germination of clostridial spores. Tumor-induced mucosal ulceration allows for translocation of sporulated bacteria from the bowel into the bloodstream, leading to fulminant sepsis.* C. septicum* bacteremia can have a variable presentation and is associated with greater than 60% mortality rate. The majority of deaths occur within the first 24 hours if diagnosis and appropriate treatment measures are not promptly started. We report a case of abdominal myonecrosis in a patient with newly diagnosed colon cancer. The aim of this study is to stress the importance of maintaining a high suspicion of* C. septicum* infection in patients with underlying colonic malignancy.

## 1. Introduction

The Clostridia species are opportunistic pathogens. Nonetheless, they are responsible for some of the deadliest diseases including gas gangrene, tetanus, and botulism. Clostridial infections were previously known to be a complication of traumatic or surgical wounds causing necrotizing skin or soft tissue infections.* Clostridium septicum* is a rare subgroup found to cause atraumatic myonecrosis, and in over 80% of cases it is associated with underlying malignancy [1]. It has been reported that the association between* C. septicum* and malignancy is due to mucosal ulceration, allowing patients with colon cancer, acute leukemia, and cyclical neutropenia to have an ideal portal of infection for the organism [2].* C. septicum* sepsis is associated with a high mortality rate, with the majority of deaths occurring within the first 24 hours [3]. We report a unique case of newly diagnosed colon cancer and subsequent development of abdominal myonecrosis to emphasize the importance of having a high suspicion for* C. septicum* in patients with malignancy. This will allow for prompt intervention with broad-spectrum antibiotics and possible surgical debridement.

## 2. Case Presentation

**Figure 1:**
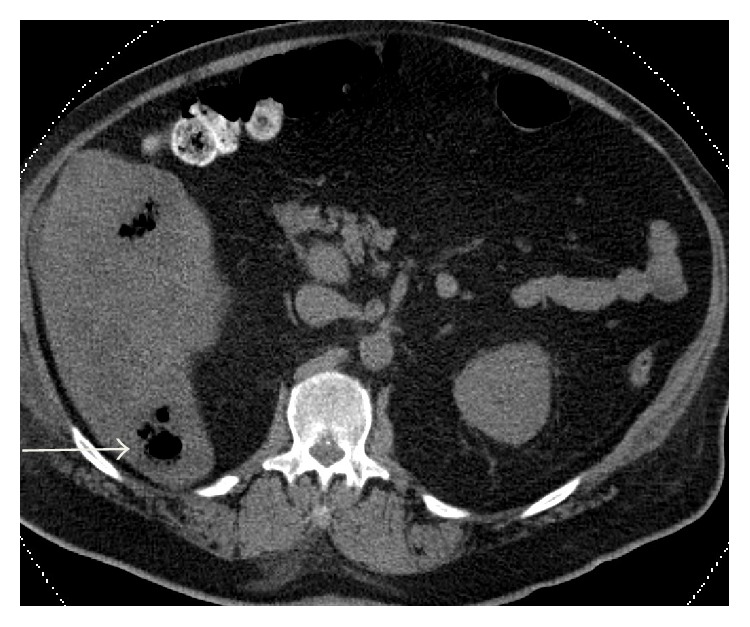


**Figure 2:**
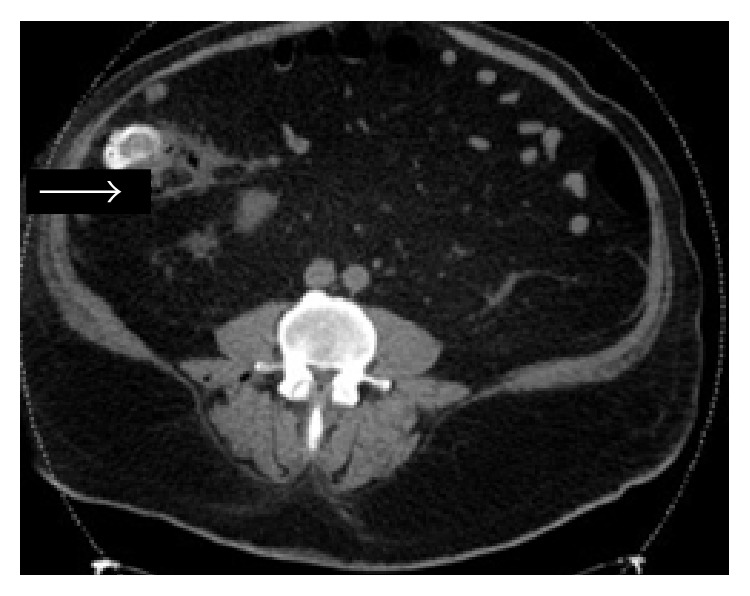


**Figure 3:**
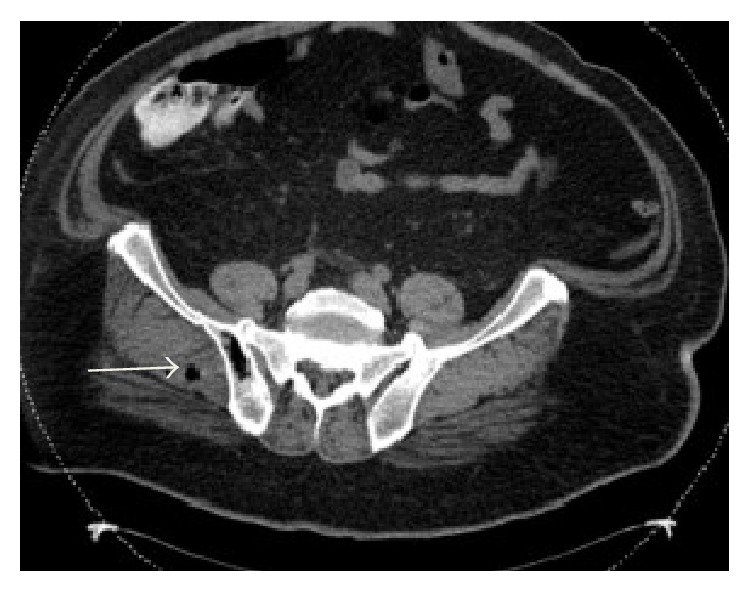


A 54-year-old male with a past medical history of hypertension presented with a ten-day history of severe bilateral lower abdominal pain radiating to his back. He reported a twenty-pound weight loss over the past six months. Upon presentation, he was afebrile and his vital signs were stable. On exam, the abdomen was diffusely tender to palpation and bowel sounds were normal with no peritoneal signs. A CT abdomen/pelvis showed multiple hepatic and ascending colonic lesions, with pericolonic fat infiltration and periportal lymphadenopathy. On day two of admission, an ultrasound guided liver biopsy was performed and pathology showed metastatic adenocarcinoma consistent with primary colonic malignancy. Patient underwent staging with a chest CT, which was negative for metastatic disease. On day three of admission, the patient became hypotensive with a blood pressure (BP) of 105/62 mmHg which was thought to be secondary to pain medications and responded well to fluid resuscitation. Subsequently, patient's lab values revealed an elevated creatinine (Cr) of 2.6 mg/dL, which was believed to be due to acute tubular necrosis secondary to hypotensive episodes. On day five of admission, the patient was persistently hypotensive with a BP of 100/60 mmHg, which did not respond to intravenous (IV) fluid resuscitation, and he was transferred to the intensive care unit to initiate therapy with vasopressors. Peripheral blood cultures were drawn, and the patient was empirically started on IV piperacillin/tazobactam 3.375 g every 6 hours and metronidazole 500 mg every 8 hours as per Infectious Disease recommendations. His lab values revealed leukocytosis of 16.4 K/*μ*L, creatinine of 4.1 mg/dL, total bilirubin of 3.3 mg/dL, AST of 407 U/L, ALT of 90 U/L, alkaline phosphatase of 259 U/L, and lactic acid of 4.1 mmom/L. Final blood cultures were positive for* C. septicum*; no anaerobic susceptibilities are performed at our hospital. The patient was continued on the initial broad-spectrum antibiotic regimen of IV piperacillin/tazobactam and metronidazole for a planned 14-day treatment. Repeat CT of abdomen/pelvis showed gas collections in the liver, peritoneum (Figures 1 and 2), multiple soft tissue, and bone (Figures 3 and 4), areas suggestive of clostridial gas gangrene. Lab work indicated worsening liver and kidney functions and the patient developed multiorgan failure. Upon discussion with the patient's family, the decision was made for comfort measures only. The patient expired on hospital day 13.

## 3. Discussion

*Clostridium septicum* was first isolated from the blood of a cow in 1877 by L. Pasteur and J. Joubert. In 1881, R. Koch proved the organism to be responsible for malignant edema, which is defined as acute, rapidly fatal toxaemia usually caused by* Clostridium* species.* C. septicum* is a Gram-positive, anaerobic, spore-forming bacillus that normally grows in soil and is a causative agent of atraumatic myonecrosis [4].* C. septicum* produces multiple exotoxins, including alpha, beta, gamma, and delta toxins. Of these, the alpha toxin is lethal, hemolytic, and necrotizing; however, unlike the alpha toxin of* C. perfringens*, the mechanism by which the alpha toxin of* C. septicum* contributes to pathogenesis is unknown. Nevertheless, it remains an important virulence factor in* C. septicum* mediated myonecrosis [5]. Although rare, in the setting of malignancy or immunosuppression, it is associated with direct, spontaneous infections of the bowel and peritoneal cavity. The anaerobic glycolysis of the tumor produces an acidic, hypoxic environment favoring germination of clostridial spores [6]. Tumor-induced mucosal ulceration causes disruption of the normal barrier, which allows for translocation of the sporulated bacteria from the bowel into the bloodstream leading to fulminant sepsis. Once the malignancy outgrows its blood supply, the anaerobic environment created is ideal for bacterial growth [7]. Mucosal disruption can also be caused by bowel perforation, surgery, radiation, or a medical procedure such as colonoscopy or barium enema. Impaired host immunity from alcohol abuse, steroids, atherosclerosis, diabetes, or neutropenia is also believed to facilitate translocation.* C. septicum* is more aerotolerant than* C. perfringens*; thus it is more likely to infect healthy tissue. The clinical spectrum of* C. septicum* varies and can present as cellulitis, fasciitis, myonecrosis, abscess, aortitis, or septic shock. However, this bacterium can also present with nonspecific symptoms including abdominal pain, fever, and malaise [8].

Clostridial infections at a single institution were reviewed to determine impact on mortality. Of the cases reviewed, 281 patients had culture proven clostridial infection and* C. septicum* was found to be the responsible species in 11.4% (*n* = 32) of cases. There was 56% mortality rate in* C. septicum* patients as opposed to 26% mortality rate in all clostridial infections. An associated malignancy was found in 50% of* C. septicum* cases, and the remaining 50% of patients had evidence of immunosuppression [9]. In another study, 241 clostridial infections were identified, of which 7.8% were* C. septicum*. There was 25% mortality rate for all clostridial infections in comparison to 80% mortality rate for* C. septicum* species alone [10].

Treatment of* C. septicum* bacteremia consists of early surgical debridement and antibiotic therapy. The empiric antibiotics of choice include IV piperacillin/tazobactam 4.5 g every 6 hours and IV metronidazole 500 mg every 8 hours. For* Clostridium* species, other appropriate antibiotics include penicillin, clindamycin, cefoxitin, ampicillin/sulbactam, and imipenem/cilastatin. The optimal duration of IV antibiotic treatment has not been defined, although treatment should continue until no further surgical debridement is needed and the patient's hemodynamic status has stabilized [11].

As previously stated,* C. septicum* is a rare and lethal diagnosis, and therefore early identification and initiation of treatment are crucial to decrease mortality. There should be a high suspicion of* C. septicum* infection in patients who present with an underlying colonic malignancy with signs of sepsis. Blood cultures should be obtained early in order to achieve a timely diagnosis [3]. In patients whom* C. septicum* infection is diagnosed without a clear underlying etiology, there should be a strong suspicion for an associated malignancy.

The best known association between bacterial infections and malignancy is* Streptococcus bovis* and colon carcinoma; however, the connection between* C. septicum* and large bowel malignancies is well demonstrated in multiple literature reviews. A review of 162 published cases of* C. septicum* infection was performed, demonstrating that 81% of patients had an associated malignancy, of which 34% had an associated colon carcinoma and 40% had an associated hematologic malignancy [2]. Therefore, in the absence of hematological malignancy, colonoscopy is warranted to evaluate colon carcinoma [6]. The majority of deaths occur within the first 24 hours if diagnosis and appropriate treatment measures are not promptly started.

## 4. Conclusion

*C. septicum* infections are strongly associated with malignancy. In septic patients with hematologic or colorectal cancer, concern for* C. septicum* bacteremia should remain high. Aerobic and anaerobic cultures should be drawn prior to starting empiric antibiotics. Early diagnosis and aggressive initiation of treatment, including antibiotics and surgical intervention, are crucial in order to improve prognosis and potentially be lifesaving in this deadly infection.

## Figures and Tables

**Figure 4:**